# Potential of montmorillonite modified by an organosulfur surfactant for reducing aflatoxin B1 toxicity and ruminal methanogenesis in vitro

**DOI:** 10.1186/s12917-022-03476-1

**Published:** 2022-11-03

**Authors:** Yosra A. Soltan, Amr S. Morsy, Nesrein M. Hashem, Mahmoud A. I. Elazab, Mohamed A. Sultan, Amr El-Nile, Haneen N. Marey, Gomaa Abo El Lail, Nagwa El-Desoky, Nourhan S. Hosny, Ahmed M. Mahdy, Elsayed E. Hafez, Sobhy M. A. Sallam

**Affiliations:** 1 Animal and Fish Production Department, Faculty of Agriculture, Alexandria University, Alexandria, Egypt; 2 Livestock Research Department, Arid Lands Cultivation Research Institute, City of Scientific Research and Technological Applications, Alexandria, Egypt; 3 Economic and Agribusiness Department, Faculty of Agriculture, Alexandria University, Alexandria, Egypt; 4 Soil and Water Sciences Department, Faculty of Agriculture, Alexandria University, Alexandria, Egypt; 5 Plant Protection and Biomolecular diagnosis Department, Arid Lands Cultivation Research Institute, City of Scientific Research and Technological Applications, Alexandria, Egypt

**Keywords:** Nano clays, Sodium dodecyl sulfate, Gas production, Sulfite, Methane, AFB1

## Abstract

**Background:**

Montmorillonite clay modified by organosulfur surfactants possesses high cation exchange capacity (CEC) and adsorption capacity than their unmodified form (UM), therefore they may elevate the adverse impact of aflatoxin B1 (AFB1) on ruminal fermentation and methanogenesis. Chemical and mechanical modifications were used to innovate the organically modified nano montmorillonite (MNM). The UM was modified using sodium dodecyl sulfate (SDS) and grounded to obtain the nanoscale particle size form. The dose-response effects of the MNM supplementation to a basal diet contaminated or not with AFB1 (20 ppb) were evaluated in vitro using the gas production (GP) system. The following treatments were tested: control (basal diet without supplementations), UM diet [UM supplemented at 5000 mg /kg dry matter (DM)], and MNM diets at low (500 mg/ kg DM) and high doses (1000 mg/ kg DM).

**Results:**

Results of the Fourier Transform Infra-Red Spectroscopy analysis showed shifts of bands of the OH-group occurred from lower frequencies to higher frequencies in MNM, also an extra band at the lower frequency range only appeared in MNM compared to UM. Increasing the dose of the MNM resulted in linear and quadratic decreasing effects (*P* < 0.05) on GP and pH values. Diets supplemented with the low dose of MNM either with or without AFB1 supplementation resulted in lower (*P* = 0.015) methane (CH_4_) production, ruminal pH (*P* = 0.002), and ammonia concentration (*P* = 0.002) compared to the control with AFB1. Neither the treatments nor the AFB1 addition affected the organic matter or natural detergent fiber degradability. Contamination of AFB1 reduced (*P* = 0.032) CH_4_ production, while increased (*P* < 0.05) the ruminal pH and ammonia concentrations. Quadratic increases (*P* = 0.012) in total short-chain fatty acids and propionate by MNM supplementations were observed.

**Conclusion:**

These results highlighted the positive effects of MNM on reducing the adverse effects of AFB1 contaminated diets with a recommended dose of 500 mg/ kg DM under the conditions of this study.

## Background

Greenhouse gases (GHG) emissions can substantially impact climate change and global warming. With the increasingly GHG, the challenge of mycotoxins, especially that of aflatoxin B1 (AFB1) would be of higher importance. Changes in the geographic distribution of mycotoxigenic fungi would also be a result of global warming [1–3]. Thus it seems that mitigation strategies of both GHG and mycotoxins are likely more suitable for the near future. The sector of livestock contributes up to 18% of the global GHG from anthropogenic origins, in which enteric methane (CH_4_) represents almost 37% of total GHG from livestock [4]. Methane is constantly increasing and it has 28 times more warming potential as a GHG than carbon dioxide (CO_2_). Therefore, CH_4_ mitigation can result in a fast cooling impact on the atmosphere [5] and may save from 3 to 12% of the dietary digestible feed energy loss [6].

The AFB1 is among the most potent hepatocarcinogenic and immunosuppressive metabolites, and also is considered the most resistant mycotoxin to ruminal microbial degradation [7], as only 10 to 50% of AFB1can be degraded by ruminal microorganisms [8]. Contaminated AFB1 diets usually exhibit symptoms including decreases in ruminal degradability and fermentation characteristics, feed intake, milk production, and growth performance [7–9]. In addition, when dairy animals consume diets contaminated with AFB1, aflatoxins M1 can be formed as a result of the metabolic process and excreted in milk [10]. Thus a significant risk can be exposed to human beings (especially children; the high milk consumers) through the consumption of contaminated milk. It seems that protecting the environment (by mitigating ruminal CH_4_ emissions) and producing safe animal products are important challenges of animal production.

Clays are generally recognized as safe for both human and animal consumption [11, 12]. Recently, modified clays exhibited higher adsorption capacity and antimicrobial activity than their raw clays [13]. Montmorillonite is one of the smectite clays, it has a 2:1 layered structure, each layer consists of two tetrahedral sheets of silicon dioxide sandwiching one octahedral sheet of aluminium oxide, these layers are separated by interlayer spacing containing various exchangeable cations [13]. Montmorillonite has high antimicrobial, aflatoxins adsorptive capacity, and buffering characteristics [14]. However, compared to other kaolinite clays, natural montmorillonite possesses lower inhibiting methanogenesis activity [11] but had higher global availability and lower cost. Therefore, it is widely used as a feed supplement to ruminant diets. Moreover, montmorillonite has a unique character, that is the high suitability to be organically modified compared to other kaolinite clays. This happens via exchanging the interlayer cations with organic cations or onions that increase the interlayer spacing between its layers, thereby enhancing its hydrophobic and adsorptive characteristics [14].

Ruminal modifiers containing sulfur or sulfate are capable of using H+ even at low concentrations to produce hydrogen sulfide (H_2_S), this action consumes eight electrons and thus can be an alternate electron acceptor to methanogenesis [15, 16]. Sulfite, in addition to inhibiting CH_4_ production by consuming H+, is toxic to methanogens [16]. Therefore, sulfonated montmorillonite (montmorillonite modified by organosulfur surfactants) may enhance the clay’s anti-methanogenic activity. Sodium dodecyl sulfate (SDS; CH_3_(CH_2_)_11_OSO_3_Na) is among the organosulfur anionic surfactant class and consists of a sodium salt of the 12-carbon an organosulfate. It is used widely to modify montmorillonite through intercalation by replacing its exchangeable cations (i.g., Ca^2+^) to change its surface properties from hydrophilic to hydrophobic, thereby increasing its adsorption capacity [17]. In our previous work, montmorillonite modified by SDS in a nano form possessed high CEC and reduced CH_4_ production by 38% when supplemented at 500 mg/ kg dry matter compared to the unsupplemented diet [12]. However, to what extent these modified clays are also operative in diets contaminated with AFB1, to the best of our knowledge, has not yet been tested empirically. Due to the high CEC of the montmorillonite, Ca^2+^ in its interlayer can be changed with ions of Na^+^ located in the structure of SDS ions, thereby increasing the intermediate distance between lamellae [17], and consequently may enhancing its affinity for aflatoxin contaminants. Furthermore, mechanical nano grinding has been proven to enhance the clay’s physicochemical, stability, and anti-methanogenic properties [18]. Therefore it was hypothesized that organo-modified nano montmorillonite by SDS (MNM) may elevate the AFB1 adverse effects while inhibiting CH_4_ formation. This is the first investigation to study the impacts of modified clays supplemented with a diet contaminated with AFB1 on ruminal fermentation and nutrient degradability.

## Methods

### Study location

This study was carried out at the Laboratory of Animal Nutrition, Department of Animal and Fish Production, Faculty of Agriculture, Alexandria University, Alexandria, Egypt.

### Preparation and characterization of the experimental clay feed additive

The commercially un-modified natural Egyptian Ca-montmorillonite clay (UM) (with 95% purity) was purchased from (Egypt Bentonite and Derivatives Co., Alex., Egypt). The experimental MNM was prepared according to the method of Bujdáková et al. [13], but with some modifications. Sodium dodecyl sulfate (SDS; Sigma Aldrich Co., Irvine, Scotland) was used as an anionic organosulfur surfactant to modify the UM clay [12]. Five g of UM clay was dispersed in 300 ml of distilled water for 24 h at room temperature using a magnetic stirrer and then the desired amount of the SDS (depending on CEC and molecular weight) was slowly added. The reaction mixture was stirred for 5 h at 80 °C. Once the cation exchange reaction occurred, the resulting organoclay suspension was stirred for 12 h at room temperature, filtered, washed three times with distilled water to remove the remains of SDS that did not interact with the montmorillonite, then dried at 90 °C. The dried clay material was grounded using a high-energy planetary ball miller (Retsch PM, VERDER SCIENTIFIC, North Rhine-Westphalia, Haan, Germany) for 5 hours, with a reverse rotation speed of 300 rpm and vial rotation speed of 600 rpm with the zirconia balls to powder ratio of 9:1 mass/mass. The clay particle size was measured by a nano-size analyzer (Malvern, Nano series, Worcestershire, UK) and recorded mean values of 98.2 ± 26.3 and 765 ± 20.9 nm for UM and MNM, respectively.

The physicochemical properties of the experimental clays were determined. The CEC of the experimental clays was analyzed according to the method of Rhoades [19] using solutions of 1 M sodium acetate and 0.1 M sodium chloride. The measured CEC for UM and MNM were 77.5 and 117 (meq/100 g), respectively. The surface charge of the experimental clays was determined by Zeta potential analysis (Malvern ZETASIZER Nano series, Worcestershire, UK) with ranges of particle size detection from 0.3 nm to 10 μm at 25.0 ± 1°C, measurement position (mm) 2.0, count rate (kcps) 347.4, and attenuator 7.0, KCl (0.150 g/100 ml solid to solution ratio) was used as an indifferent electrolyte. The measured Zeta potential values were − 23.1 and − 24.0 mV for UM and MMM, respectively.

The nanoparticle’s shape of MNM was recorded by using a scanning electron microscope (SEM; Jeol JSM-6360 LA, 3–1-2 Musashino, Akishima, Tokyo, Japan) after coating with gold to improve the imaging of the clay sample [18]. The functional groups of the experimental clays were determined by Fourier Transform Infra-Red Spectroscopy (FTIR) by an infrared spectrometer (Shimadzu-8400S, Osaka, Japan) as described by Soltan et al. [12]. The FTIR analysis was performed on a detector of deuterated triglycine sulfate and a beam splitter of KBr. The scanning rate is 45 scans/ 60 seconds, and the mass ratio of the clay sample to KBr was 1 mg of KBr and 99 mg of clay.

The *d*-spacing of the UM and OMNM clays was characterized by X-ray diffraction (XRD) using a MeasSrv (D2–208219/ D2) powder diffractometer with CuKα radiation filtered with a graphite monochromator that running at 40 kV and 40 mA as described by Elshazly and Hamdy [20]. The XRD had a fixed source-sample-detector geometry, and samples were measured in reflection mode. An X-ray diffraction data set was collected from 1 to 60 2θ. The tilt angle between the source and the sample was 5.8, and the horizontal slit system was set at 0.14 mm to confine the x-ray beam to pure cobalt Kα1.

### In vitro gas production (GP) assay

#### The experimental basal diet

A 500: 500 forage to concentrate was used as an experimental basal diet, the forages were the traditional Egyptian berseem clover hay (*Trifolium alexandrinum*) and wheat straw. This diet was prepared to fulfill the national research council’s nutrient requirements for dairy sheep [21]. The primary ingredients and chemical composition of this diet are presented in Table 1. The basal diet was chemically analyzed following the Association of Official Analytical Chemists [22] for DM, organic matter (OM), crude protein (CP), and ether extract (EE). Fiber contents of neutral detergent fiber (NDF), acid detergent fiber (ADF), and acid detergent lignin (ADL) were sequentially analyzed according to Van Soest et al. [23] using a semi-automatic fiber analyzer (ANKOM, model A2001, Macedon, NY, USA) in filter bags (F57- ANKOM Technology Corporation, Macedon, NY, USA).

**Table 1:** Ingredients and chemical composition based on dry matter (DM) of the experimental basal diet

Item	Experimental diet
	(g/kg DM)
Ingredients	
*Trifolium alexandrinum* clover	350
Wheat straw	150
Ground maize	225
Wheat bran	165
Soybean meal	90
Calcium carbonate	10
Sodium chloride	5
Vitamins and minerals mixture^a^	5
Chemical composition	
Organic matter	923
Crude protein	138
Neutral detergent fiber	425
Acid detergent fiber	214
Acid detergent lignin	49.8
Ether extract	35.1

^a^ Contaning (per kg) manganese 80 mg, zinc 60 mg, iron 35 mg, copper 8 mg, selenium 0.6 mg, choline chloride 600 mg, vitamin B6 3 mg, thiamine 3 mg, folic acid 1.0 mg, d-biotin 50 μg, Ca-pantothenate 1 mg, nicotinic acid 50 mg, menadione 1.3 mg, riboflavin 5.5 mg, vitamin B12 10 μg,.vitamin A 12,000 International Unit (IU), vitamin D3 2500 IU and vitamin E 20 IU

The total aflatoxins (AFs) of the basal diet were extracted and purified in duplicate by VICAM immunoaffinity columns (VICAM Aflatest, Milford, MA, USA) as described by Hafez et al. [24] and quantified using the VICAM fluorometry method (VICAM Series 4EX Fluorometer, Milford, MA, USA) according to the manufacturer’s instructions [25]. We recorded values of 13.8 and 14.4 ppb (an average of 14 ppb) AFs in the basal diet without any supplementations.

#### Treatments and GP protocol

Eight treatments were evaluated in vitro to test the dose-response effects of the MNM supplemented on the basal diet contaminated or not with a final concentration of AFB1 20 ppb (produced by *Aspergillus flavus,* 98% purity, Sigma Chemical Co, Louis, Missouri, USA) using the semiautomatic gas production system. The treatments were: control (basal diet without clay supplementations), unmodified-montmorillonite (UM) supplemented at 5000 mg /kg DM, and OMNM diets at low (500 mg/ kg DM) and high doses (1000 mg/ kg DM). The final AFs concentrations of the basal diet contaminated with AFB1 were 34 ppb. These doses are higher than the Egyptian maximum permissible concentrations for the AFs and AFB1 (20 ppb and 10 ppb, respectively) for dairy animal feeds [26].

The treatments were evaluated using the semi-automatic gas production system as described by Bueno et al. [27] and adapted by Soltan et al. [28]. To prepare the ruminal inoculum for the in vitro assay, ruminal contents were collected separately from three fasted, slaughtered crossbred buffalo calves (450 ± 7 SE kg body weight) from the slaughterhouse of the farm station of the Faculty of Agriculture, Alexandria University, Egypt [29].

The ruminal contents were transferred immediately into pre-warmed thermo-containers (39 °C) under carbon dioxide (CO_2_) flushing. The ruminal inoculum was prepared by blending the ruminal contents of slaughtered calves in equal proportions (1:1:1) for 10 s, then squeezed by three layers of cheesecloth, and kept in a water bath (39 °C) under continuous flushing of CO_2_. Twelve in vitro incubation glass bottles (120 ml; Arab Pharmaceutical Glass Company, Suez, Egypt) were prepared for each treatment.

An amount of 500 mg of each experimental diet was weighed into an incubation bottle and incubated with 15 ml of the prepared ruminal inoculum and 30 ml of Menke’s buffer solution, thus the headspace was 75 ml [12, 28]. The incubation bottles were tightly closed by 20 mm butyl rubber stoppers and sealed with aluminum seals. The incubation was done for 24 h at 39 °C in a forced air incubator (FLAC STF-N 52 Lt, Lombardy, Italy). The same process was done for blank bottles (containing buffer solution and ruminal inoculum) and internal standard bottles (containing buffer solution, ruminal inoculum, and the Egyptian berseem clover hay) to get the net G*P* values and correct for sensitivity variations induced by the inocula, respectively.

### Experimental parameters

#### Gas and CH_4_ productions

The gas pressure of the incubation bottles was recorded at 3, 6, 9, 12, and 24 h from the start of the incubation using a pressure transducer and data logger (Pressure Press Data GN200, Sao Paulo, Brazil). The GP volumes (ml) were calculated as 4.97 × measured gas pressures (psi) + 0.171 (*n* = 500 samples; r^2^ = 0.99) [28].

To determine the CH_4_ production, one ml of the headspace gas of each bottle was sampled at each gas pressure measuring time by a 3 ml syringe and was accumulated in a 5 ml vacutainer tube (Vacutainer® Tubes, Jersey, USA). Concentrations of CH_4_ were determined by gas chromatography (GC, Agilent GC Analyzers Greenhouse Gas Analyzer, with Power Supply of 2 kW) provided by Agilent Technologies, Inc., Santa Clara, California, USA.

#### Ruminal fermenattion parametrs and protozoal count

At the terminal of the incubation period, all the incubation bottles have been set on ice to stop the ruminal microbial actions. Values of pH were measured by a pH meter (CRISON GLP2, Barcelona, Spain). The ammonia concentrations were determined using a commercial kit (Biodiagnostic Inc., Giza, Egypt). The concentrations of short-chain fatty acids (SCFAs) were measured following Palmquist and Conrad [30] using gas chromatography (GC; Scion 456-GC/FID, Netherland). The GC was equipped with a capillary Rt-2560 column (100 m × 0.25 mm ID, 0.20 μm df, Restek) with a constant flow of 1.2 ml/ min helium as carrier gas. A SCFAs standard mix (Sigma Aldrich Co., Irvine, Scotland) was used to obtain absolute quantification of each SCFAs.

The total protozoal count was counted through microscopy as described by Dehority et al. [31] using a bright-line hemacytometer counting chamber (Paul Marienfeld Gmb H & Co. K.G., Baden-Württemberg, Germany).

#### Ruminal nutrient degradability

The contents of the incubation bottles were treated with the neutral detergent solution for 3 hours at 90 °C to determine the nutrient degradability Blümmel et al. [32]. The residuals non-degraded of the contents of the bottles were collected in pre-weighed free crucibles, washed with hot distilled water and acetone, dried at 70 °C for 48 hrs, and then ashed at 600 °C for 2 hrs. The truly degraded organic matter (TDOM) and truly degraded neutral detergent fiber (TDNDF) were calculated by the differences between the incubated and non-degraded organic matter amounts and between the amount of incubated NDF and the non-degraded NDF amounts, respectively [28].

### Statistical analysis

The in vitro experiment was completed in one run (1 day) for all the experimental diet treatments. The experimental unit was the incubation bottle, thus 12 statical repetitions were obtained for each treatment. All results were analyzed by one-way ANOVA using the MIXED procedure of SAS [33] (SAS Institute Inc., Cary, USA, version 9.0). The experimental parameters were processed as a completely randomized design with repeated measures using the following model:

Yijkl = μ + Di + Tj + Iik + (D × T)ij + eijkl, where Yijkl the observation; μ was the overall mean; Di fixed effect of experimental diet; Tj fixed effect of AFB1 supplementation; Iik random effect of the diets; (D × T) ij interaction effect between diet and AFB1 supplementation, and eijkl the residual error. In addition, orthogonal contrast statements were designed to test linear and quadratic responses of each dependent in vitro parameter to the increasing levels (0, 500, 1000 mg/kg DM) of OMNM. The effects were declared significant at *P* ≤ 0.05, and the trends at *P* ≤ 0.10.

## Results

### Physicochemical properties of the experimental clays

**Fig. 1:**
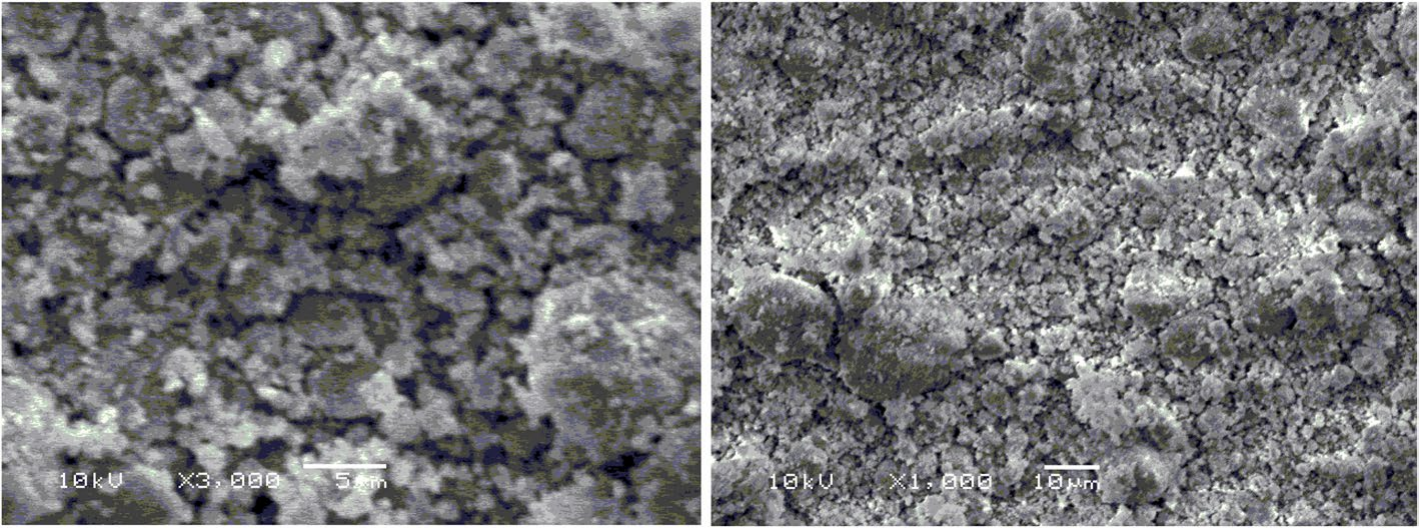
The surface morphology of the modified nano montmorillonite (MNM) by a scanning electron microscope

**Fig. 2:**
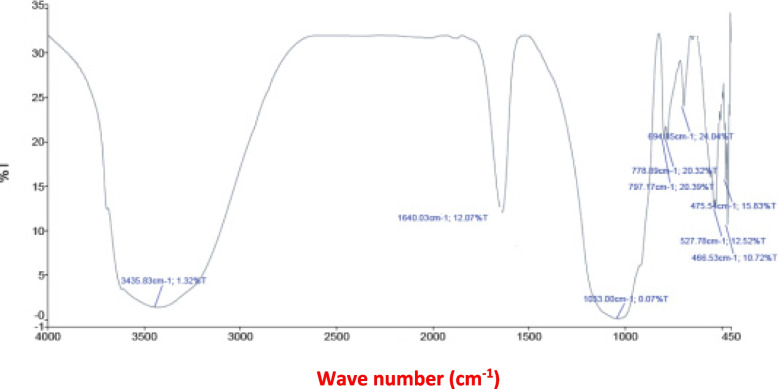
Fourier transform *infrared spectroscopy* (FTIR) analysis for the modified nano montmorillonite (MNM)

**Fig. 3:**
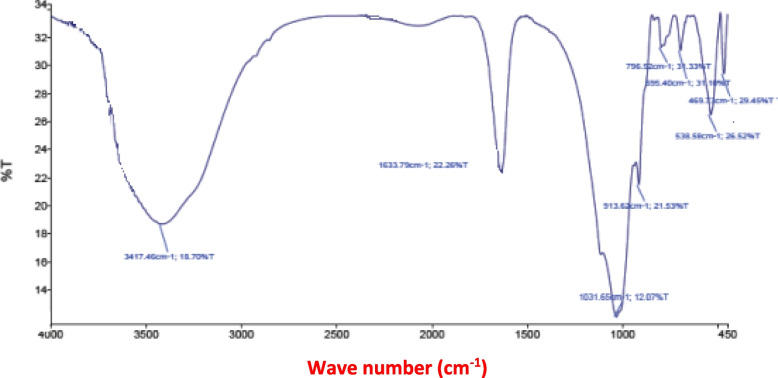
Fourier transform *infrared spectroscopy* (FTIR) analysis for the un-modified montmorillonite (UM)

The SEM micrographs of the modified clay are illustrated in Fig. 1. The SEM analysis showed a cracked and rough appearance of the MNM. The FTIR bands of the experimental clays are represented in Table 2 and Figs. 2 and 3. Bands of the OH-group were shifted from 3417 cm^− 1^ in UM to higher frequencies at 3435 cm^− 1^ in MNM, also for the medium bands, they shifted from lower frequency at 1633 cm-1 in UM to higher frequency 1640 cm-1 in MNM. Only for the modified clay, a band at 778 cm-1 appeared which attributed to the Si–O stretching vibrations, while it was absent in the UM clay. In the lower frequency range (450 and 550 cm-1), an extra band at 475 cm^− 1^ only appeared just in MNM.

**Table 2:** Fourier transform *infrared spectroscopy* (*FTIR*) analysis of unmodified montmorillonite (UM) and modified nano montmorillonite (MNM)

Peak number	Maxima (cm − 1)	
	UM	MNM
1	3417	3435
2	1632	1640
3	1031	1033
4	913	797
5	796	778
6	695	694
7	538	527
8	469	475
9		466

**Fig. 4:**
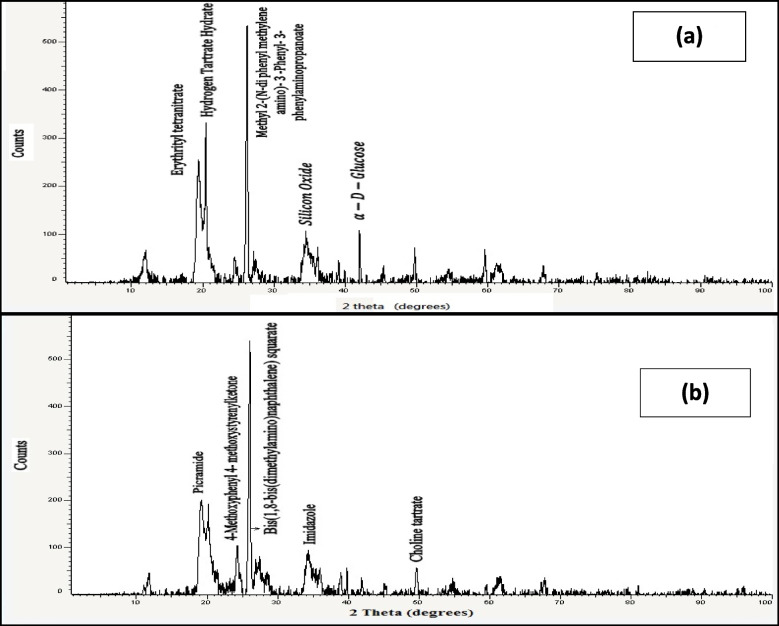
X-ray diffraction (XRD) pattern for the modified nano-montmorillonite (MNM) (**a**) and natural un-modified montmorillonite (UM) (**b**)

The XRD patterns of UM and MNM clay are presented in Fig. 4. The XRD spectrograms showed that UM consists mostly of picramide or 2,4,6-trinitroaniline (48.2%) and bis(1,8-bis (dimethylamino)naphthalene) squarate (19%), while MNM clay consists mostly of methyl 2-(N-diphenylmethylene amino)-3-phenyl- 3- phenyl amino propanoate (39.2%), and erythrityl tetranitrate (40.3%).

### Gas and CH_4_ production, and nutrient degradability

Results in Table 3 showed that MNM modified by SDS affected GP and CH_4_ productions differently compared to the unmodified clay. Treatment, MNM dose, and MNM dose × AFB1 interaction affected (*P* < 0.01) the GP values, while no effects were detected by AFB1 supplementation. Among the experimental treatments, diets supplemented with MNM generally resulted in lower (*P* < 0.01) GP values than the UM and control diets. The control diet contaminated with AFB1 had lower (*P* < 0.01) GP than the control diet without supplementations. Concerning the MNM dose effect, the high level showed lower (*P* < 0.01) GP values compared to the low level. The contrast analysis showed also significant effects (*P* < 0.05) for the MNM addition, since the increasing dose of the MNM resulted in linear and quadratic decreasing effects (*P* < 0.05) GP values.

**Table 3:** Supplementation effects of unmodified montmorillonite (UM) and modified nano-montmorillonite by sodium dodecyl sulfate (MNM) on ruminal gas production (GP), methane emission (CH_4_), true degraded organic matter (TDOM), and true degraded neutral detergent fiber (TDNDF)

Item	Treatments (T)									*P* value					
	Control		UM		MNM				SEM		MNM				
					Low		High							Contrast	
	-AFB1	+AFB1	-AFB1	+AFB1	-AFB1	+AFB1	-AFB1	+AFB1		T	Dose	AFB1	Dose×AFB1	Linear	Quadratic
GP (ml/ g DM)	164	141	126	120	133	127	127	119	3.21	<.0001	<.0001	0.251	<.0001	<.0001	0.013
CH_4_ (ml/ g TDOM)	26.4	21.8	21.7	22.1	20.5	21.6	24.1	21.6	0.73	0.015	0.030	0.032	0.051	0. 242	0.016
Nutrient degradability															
TDOM	771	762	779	768	786	783	746	715	10.6	0.562	0.211	0.544	0.879	0.240	0.180
TDNDF	448	426	466	439	485	476	388	311	18.4	0.263	0.201	0.301	0.502	0.782	0.223

*SEM* standard error of the mean, Low = MNM supplemnted at 500 mg/ kg dry matter, High = MNM supplemnted at 1000 mg/ kg dry matter Contrast: = effects of control (0 supplementation mg/kg dry matter) compared with low and high MNM

Among the experimental treatments, diets supplemented with the low dose of MNM either with or without AFB1 supplementation resulted in lower (*P* = 0.015) CH_4_ production compared to the control without AFB1. Similarly, the MNM does, AFB1 and dose × AFB1 interaction affected significantly (*P* < 0.05) the CH_4_ production. Methane produced by diets supplemented with low MNM does was declined (*P* = 0.030) compared to the control or high dose. AFB1 also resulted in a CH_4_ decreasing (*P* = 0.032) effect compared to the non-supplemented diets. The contrast analysis showed that the decrease in CH_4_ (related to TDOM) by MNM was in a dose-dependent manner; MNM reduced CH_4_ in a quadratic (*P* = 0.016) trend. Neither treatment nor AFB1 or MNM affected the TDOM and TDNDF.

### Rumianl fermentaion parametrs and protozaol count

The effects of the feed additives on ruminal pH, ammonia, protozoa, and SCFAs are shown in Table 4. Treatment of UM either with or without AFB1 resulted in a decrease (*P* = 0.02) of ruminal pH values compared to the control. Contamination of AFB1 increased (*P* = 0.02) the ruminal pH compared with non contaminated diets. A linear reduction effect (*P* = 0.01) of ruminal pH was observed by the MNM supplementation.

**Table 4:** Supplementation effects of unmodified montmorillonite (UM) and modified nano-montmorillonite by sodium dodecyl sulfate (MNM) on ruminal pH, ammonia concentrations, total short-chain fatty acids (SCFAs) concentration, and molar proportions of individual SCFAs (% of total SCFA)

Item	Treatments (T)									*P* value					
	Control		UM		MNM				SEM	T	OMNM				
					Low		High							Contrast	
	-AFB1	+AFB1	-AFB1	+AFB1	-AFB1	+AFB1	-AFB1	+AFB1			Dose	AFB1	Dose× AFB1	Linear	Quadratic
Ruminal pH	5.92	5.94	5.84	5.87	5.85	5.91	5.87	5.90	0.01	0.002	0.013	0.015	0.676	0.010	0.086
Ammonia (mg /100 ml)	18.4	27.5	19.6	22.5	15.9	19.3	19.3	25.6	2.53	0.002	< 0.001	< 0.001	0.188	0.633	< 0.001
Protozoa (10^5^/ ml)	5.90	6.05	5.80	6.40	4.40	5.80	6.30	7.00	0.06	0.471	0.164	0.248	0.716	0.390	0.089
SCFAs															
Total (mmol)	63.4	63.1	64.9	65.4	68.59	70.8	61.8	63.9	1.99	0.199	0.014	0.457	0.814	0.08	0.012
Acetate (% of total)	59.7	60.1	58.4	58.9	59.3	59.8	58.9	58.8	0.26	0.006	0.010	0.295	0.553	0.003	0.552
Propionate (% of total)	19.5	19.7	19.7	20.1	20.1	20.0	19.8	19.0	0.14	0.007	0.011	0.208	0.053	0.229	0.004
Butyrate (% of total)	12.0	11.88	12.9	12.4	12.4	12.4	12.6	12.5	0.20	0.044	0.029	0.604	0.862	0.011	0.438
Isobutyrate (% of total)	1.93	1.73	2.00	1.77	1.72	1.74	1.86	2.10	0.04	0.007	0.020	0.771	0.041	0.079	0.020
Valerate (% of total)	1.58	1.51	1.627	1.52	1.45	1.35	1.62	1.68	0.03	0.003	<.0001	0.251	0.119	0.017	<.0001
Isovalerate (% of total)	2.17	2.14	2.24	2.22	2.12	1.97	2.20	2.47	0.02	<.0001	<.0001	0.191	<.0001	<.0001	<.0001
Acetate: propionate	3.05	3.04	2.95	2.93	2.94	2.97	2.97	3.08	0.02	0.401	0.090	0.164	0.255	0.661	0.034

*SEM* standard error of the mean, Low = MNM supplemnted at 500 mg/ kg dry matter, High = MNM supplemnted at 1000 mg/ kg dry matter Contrast: = effects of control (0 supplementation mg/kg dry matter) compared with low and high MNM

Treatment, MNM dose, and AFB1 supplementation affected (*P* < 0.05) ruminal ammonia concentration, while no effects were detected by MNM dose × AFB1 interaction. Among the treatments, the control diet with AFB1 had the highest (*P* = 0.002) ammonia values, while that treated with MNM without AFB1 had the lowest values. The low MNM dose presented lower (*P* < 0.01) ammonia concentrations than the high MNM dose, similarly, AFB1 resulted in increasing (*P* < 0.01) ammonia concentration compared to diets without AFB1. A quadratic increasing effect (*P* < 0.01) was observed by the MNM supplementation on ammonia concentration. No differences were detected in the protozoal counts either by MNM or AFB1 supplementations.

Treatment and MNM dose affected (*P* < 0.05) all the SCFAs individual molar proportions, while no effects were detected by AFB1 supplementation. Significant (*P* < 0.05) interaction effects (MNM dose × AFB1) were observed on the butyrate and branched-chain volatile fatty acids (BCVFA; e.g., isobutyrate and isovalerate) molar proportions. The low MNM dose resulted in higher (*P* < 0.05) total SCFAs concentration, acetate, and propionate, and lower (*P* < 0.05) BCVFA molar proportions than the high MNM dose. These were in line with the contrast analysis since increases in acetate (linearly; *P* = 0.003), propionate, and total SCFAs (quadratically; *P* < 0.05) were observed by MNM supplementation. Similar high (*P* = 0.007) propionate molar proportions were observed in the low MNM diets with or without AFB1 and the UM diet with AFB1.

## Discussion

The measured physicochemical properties of the resultant modified MNM differed from the UM. The literature confirms the distinctive feature of UM clay is the soft and tight layers’ edges of the flakes [34, 35]. In the current study, the analysis of SEM showed that the edges of the MNM flakes became cracked and had a rough appearance after modification by SDS. This mainly happened due to the separation between the clay layers caused by the settled locations of SDS between octahedral alumina layers between two silica tetrahedron layers [35]. Similar to our findings observed by Bayram et al. [35], when modified UM by SDS but without the nano grinding). Thus it can be suggested that the nano grinding did not affect the localization of SDS between the octahedral layers. The measured CEC of the UM was high (77.5 meq/100 g) and became higher after modifications (117 meq/100 g) for the MNM clay confirming the exchange of the ions between the UM clay with our experimental ionic surfactant [17, 35]. It was proved that ions of Na^+^ located in the structure of SDS can be changed with ions of Ca^2+^ in the UM interlayer, thereby SDS anions can enter the interlayer space of Na^+^ and Ca^2+^ of UM as counter ions [17]. Bayram et al. [35] reported that the cracked appearance of montmorillonite only appeared with the presence of sulfur (2.83%) the interlayer structure of the modified clay by SDS, while it was absent in the UM clay. Therefore, it can be suggested that our MNM successfully contained sulfonate groups (RSO_3_^−^), in other words, the experimental montmorillonite (UM) became sulfonated montmorillonite (MNM). The analysis of FTIR may partly confirm our suggestion since a band located at around 475 cm^− 1^ corresponding to S − S stretching bonds [36] only appeared in MNM, while it was absent in UM. However, no extra bands crossbanding to S=O bonds were detected in MNM but regions of the medium and high bands that correspond to sulfonate and hydroxyl H–O–H bond groups were shifted from lower frequency in UM to higher frequency in MNM. Rather than, a band at 778 cm-1 appeared which was attributed to the Si–O stretching vibrations, while it was absent in the UM clay. Thus it can be suggested that anions (SO^3^) of SDS might be adsorbed and contributed to the high negative observed by MNM [35]. The high negative charges indicated by the Zeta potential of MNM may partly confirm such a suggestion. A similar profile of FTIR analysis was observed by Soltan et al. [12] that may confirm the efficiency of the modification process in producing MNM.

The results of the FTIR analysis were compatible with the higher CEC found for MNM than the UM, which indicated the high number of metal hydrolysates and ions that can be intercalated into the MNM interlayer space improving the clay activity compared to UM [12].

The XRD analysis allows the determination of phases and crystallographic properties of any experimental materials [17], it is based on each crystal phase breaking the X-rays transmitted onto it in a specific characteristic order, depending on their unique atomic sequences as a kind of fingerprint [35]. The XRD results in the current study showed changes in the crystalline structure of the montmorillonite after the modification by SDS since different crystals were observed by the two experimental clays. These results also confirmed that the MNM is a different product with different physicochemical properties from the parent clay. Recently, the compounds containing S − S bonds exhibited high biological activities like antimicrobial, antitumor, antifungal, and cytotoxic activities [36] therefore it would be expected that both UM and MNM can affect the ruminal microbial fermentation profiles differently.

Based on the results of the in vitro GP experiment, it appears both experimental clays can inhibit the GP compared to the control, but MNM generally was more efficient to reduce GP values than the UM in a dose-dependent manner. The UM is known to have an ability for capturing CO_2_ (the major component of GP) through a reaction between CO_2_ molecules and UM interlayer –OH functional groups to form –HCO^3^, which can in turn react with more interlayer cations thereafter [37]. The higher reduction in GP caused by MNM may indicate the higher absorbance efficiency to capture CO_2_ than the UM. The increases in interlayer spacing, hydrophobic surface, and CEC in addition to the observed shifts in the frequency of the hydroxyl bonds found by FTIR analysis of MNM compared with UM may enhance the absorptive efficiency of MNM to capture CO_2_ and thus reduce GP [12].

It is worth noticing that the control diet supplemented with AFB1 produced lower GP than the control diet without AFB1, it seems that AFB1 has an inhibitory effect on some rumen microorganisms [9]. The literature showed inconsistency in the impact of AFB1 on GP values. Khodabandehloo et al. [9] reported that AFB1 at concentrations up to1.5 μg/ml did not affect GP values, while when added at higher doses (5 and 10 μg/ml), a significant reduction in GP was noted. Similarly, Mojtahedi et t al [38]. reported that increasing the addition level of AFB1 from 0 to 900 ng/ml decreased GP from 196 to 166 ml/g DM, respectively, while no effects on GP were observed by Jiang et al. [39] when used AFB1 at similar concentrations. Most of these studies even did not mention the AFs concentration in the diets before AFB1 addition, which can affect the obtained results. Thus these contradictions may mainly be due to the difference in aflatoxin source, ruminal inocula, or animal diet, in addition to the AFB1 experimental doses.

The low MNM supplemented to diet with or without AFB1 presented promising inhibition in CH_4_ production without adverse effects on OM or fiber degradability compared to the control without AFB1. Hydrogen (H+) is the main metabolite in the microbial degradation of OM and NDF that methanogens mainly use to reduce CO_2_ into CH_4_ [5]. Thus, results suggested that the reductions in GP or CH_4_ by the low MNM were not a result of the general inhibition of the microbial activities, but might be specifically related to the methanogenesis process. However, this possibility has yet to be proved, but the shifts of the absorption bands of the hydroxyl and sulfate groups in the high-frequency range in the FTIR analysis in addition to the high CEC of the MNM compared to UM would indicate the higher ability to bind the H+. Moreover, the presence of a band of S-S of MNM may also interfere in the anti- methanogenesis processes. Components containing sulfur and sulfate are capable of using H+ at low concentrations to produce hydrogen sulfide (H_2_S), thereby combating methanogens to produce CH_4_ [15, 16]. Reducing sulfite to produce H_2_S consumes eight electrons and thus can be an alternate electron acceptor as well as nitrate [15]. Sulfite, in addition to inhibiting CH_4_ production, is toxic to ruminal bacteria and methanogens [16]. Methane was determined per unit of the TDOM in the current study, however, no significant differences were detected in the TDOM among the experimental treatments, but the outstanding of the low MNM dose to reduce CH_4_ compared to the high dose might be due to the numerical decreases in TDOM happened by the high MNM. Recently, Soltan et al. [12] found that supplementation of MNM modified by SDS at 500 mg/kg DM to a basal diet containing lower NDF (395 g/ kg DM) reduced CH_4_ (38%) production at a higher value than what we obtained in the current study (22.3%). In both studies, the MNM had similar physiochemical properties except for the higher particle size of the current MNM (98.2 nm) than that was used by Soltan et al. [12] (59.8 nm). It seems that reducing CH_4_ emission was primarily induced by the diet forage type (fiber content) and MNM particle size. Methane reduction by low MNM was combined with increases in propionate concentration either with or without AFB1 contamination compared to the control diet contaminated with AFB1. These results indicated that MNM may elevate the adverse effects of AFB1 on ruminal fermentation, as the affinity of MNM to cationic matters might improve its AFB1adsorption capacity. Enhancing propionate production might partly explain the reduction in CH_4_, as it also served as an alternative hydrogen sink in the rumen [40].

The addition of AFB1 reduced CH_4_ production, no clear explanations can be provided here. Studies of AFB1 effects on CH_4_ emission are rare, however, most AFB1 studies concur that it has selective inhibitory effects against ruminal microorganisms including cellulolytic bacteria [9], however, AFB1 did not affect the TDNDF in the current study. The total protozoal count was not affected by the clay treatments or AFB1, may an incubation period of 24 h in vitro assay was not an adequate time to reveal the effect of these additives on the protozoal count. It can be suggested that protozoa did not interfere in the CH_4_ reduction achievements in this study, this is because protozoa and methanogens are in a synergistic relationship, as the former can provide the required metabolites (including H_2_) for methanogenesis [41].

The effects of the experimental clays and AFB1 additions on ruminal pH can be attributed to ammonia production. Decreases in the pH by UM were combined with decreases in ammonia concentration, while the pH increases observed in AFB1 contaminated diets were combined with ammonia increases. Ammonia is the end-product of ruminal protein fermentation, thus high ammonia concentration is an indicator of the high degradation of dietary protein [41]. However, no differences were detected neither AFB1 nor MNM and UM on the TDOM, but the reductions observed in ammonia concentration were combined with reductions in BCVFA (the end products of amino acid deamination in the rumen) by low MNM dose. The presence of the acidic functional groups (SO_3_^−^) of MNM rather than the clay pore structure might enhance ammonia capture capacity in MNM [12,16]. This partly explains the reduction in ammonia by the low dose of MNM, but unexpectedly, the high supplementation level of MNM resulted in increases in ammonia and BCVFA concentrations. It seems that not only the MNM capture capacity for ammonia can be the sole indicator of rumen ammonia reduction. The balance between ammonia and BCVFA releases and uptakes by the specific rumen microbes may also affect their final concentrations [42]. It seems that the high dose of MNM adversely affected the growth of specific microbes that consume ammonia and BCVFA. This together with the decreases in GP, total SCFA, and acetate concentrations observed by the high MNM might confirm such a suggestion. Moreover, ruminal bacterial cells are known to have negatively charged sites, thus the modified montmorillonite might bind them through extracellular polysaccharides of the bacterial cell wall due to the presence of positively charged interlayer ions (e.g., Na^+^) in the clay [11]. These findings suggested that great attention has to be taken into consideration to select the proper dose of MNM to obtain the most nutritional benefits from its addition.

The results of this study are consistent with those of Khodabandehloo et al. [9] who observed high ammonia concentration in AFB1-contained cultures due to the decreased growth of cellulolytic bacteria, rumen proteolytic activity, or high microbial lysis caused by AFB1. In the current study, we only measured the total protozoal count, thus quantification information of the cellulolytic, proteolytic, or sulfate-reducing bacteria would be needed to explain our results, this is the major limitation of this study, that has to be considered in further MNM in vitro/in vivo studies.

## Conclusion

The modified nano montmorillonite by SDS exhibited exceptional physicochemical properties compared to the unmodified clay (UM), like high cation exchange capacity which might improve its adsorption capacity. Diets contaminated with AFB1 adversely affected the ruminal fermentation process, while MNM can stimulate them. Supplementation of MNM at 500 mg/kg reduced CH_4_ with increases in propionate concentration either with or without AFB1 contamination compared to the control diet contaminated with AFB1. The consideration of MNM as a ruminal fermentation modifier was dose-dependent since the high MNM supplementation dose adversely affected the ruminal fermentation profile. It seems that modifications of the clays are a new potential approach as feed additives to elevate the adverse effects of mycotoxins while reducing the GHG emission from the livestock sector, but such in vitro experiments did not account for the absorption of AFB1 and/or its metabolites into the bloodstream after ingestion. Therefore the modified clays are required to assess in vivo (with various diet types) for recommendations and practical applications.

## Data Availability

Datasets generated and/or analyzed during this study are included in this article version, and if required any further information related to the data involved in the manuscript can be obtained from the corresponding author upon reasonable request.
